# Proteomic Analysis of Pleural Effusions from COVID-19 Deceased Patients: Enhanced Inflammatory Markers

**DOI:** 10.3390/diagnostics12112789

**Published:** 2022-11-14

**Authors:** Ali Razaghi, Attila Szakos, Marwa Alouda, Béla Bozóky, Mikael Björnstedt, Laszlo Szekely

**Affiliations:** 1 Division of Pathology, Department of Laboratory Medicine, Karolinska Institute, SE-141 86 Stockholm, Sweden; 2 Laboratory of Clinical Pathology and Cytology, Karolinska University Hospital, SE-141 86 Stockholm, Sweden

**Keywords:** autopsy, COVID-19, cytokine storm, hypoxia, inflammation, JAK/STAT

## Abstract

Critically ill COVID-19 patients with pleural effusion experience longer hospitalization, multisystem inflammatory syndrome, and higher rates of mortality. Generally, pleural effusion can serve as a diagnostic value to differentiate cytokine levels. This study aimed to evaluate the pleural effusions of COVID-19 deceased patients for 182 protein markers. Olink^®^ Inflammation and Organ Damage panels were used to determine the level of 184 protein markers, e.g., ADA, BTC, CA12, CAPG, CD40, CDCP1, CXCL9, ENTPD2, Flt3L, IL-6, IL-8, LRP1, OSM, PD-L1, PTN, STX8, and VEGFA, which were raised significantly in COVID-19 deceased patients, showing over-stimulation of the immune system and ravaging cytokine storm. The rises of DPP6 and EDIL3 also indicate damage caused to arterial and cardiovascular organs. Overall, this study confirms the elevated levels of CA12, CD40, IL-6, IL-8, PD-L1, and VEGFA, proposing their potential either as biomarkers for the severity and prognosis of the disease or as targets for therapy. Particularly, this study reports upregulated ADA, BTC, DPP6, EDIL3, LIF, ENTPD2, Flt3L, and LRP1 in severe COVID-19 patients for the first time. Pearson’s correlation coefficient analysis indicates the involvement of JAK/STAT pathways as a core regulator of hyperinflammation in deceased COVID-19 patients, suggesting the application of JAK inhibitors as a potential efficient treatment.

## 1. Introduction

Despite the onset of vaccinations, the emerging new mutants of severe acute respiratory syndrome Coronavirus 2 (SARS-CoV-2) show immune-evading characteristics which may lead to potential ineffectiveness of the vaccines against new variants, posing a continuous global threat to world health and the economy [1]; thus, it necessitates ever-evolving research in the Coronavirus Disease 2019 (COVID-19) area.

Pathologically, severe COVID-19 cases are characterized by hypoxia and ravaging cytokine storms that produce an excessive immune and inflammatory response, particularly in the lungs, resulting in acute respiratory distress (ARDS), pulmonary oedema, and multiorgan failure. Pro-inflammatory factors have been identified as one of the main factors contributing to worse outcomes in COVID-19, playing an important role in severity, particularly in patients with comorbidities [2]. “Cytokine storm”, mostly detected in the peripheral blood of COVID-19 patients [3], runs with high levels of components such as interferons (IFNs), chemokines, interleukin (IL)-6, IL-8, IL-1β, and chemokines, along with reactive oxygen species, which are harmful by inducing cellular necrosis, diffusing alveolar lesion, pulmonary fibrosis, and fibrin deposition [4,5]; thus, alleviating hyperinflammation is crucial to improving prognosis. Moreover, T and B lymphocytes play crucial roles in COVID-19 severity and defence; for example, according to one study, increased T lymphocyte exhaustion and decreased T lymphocyte function may predict severe COVID-19 cases [1].

**Figure 1:**
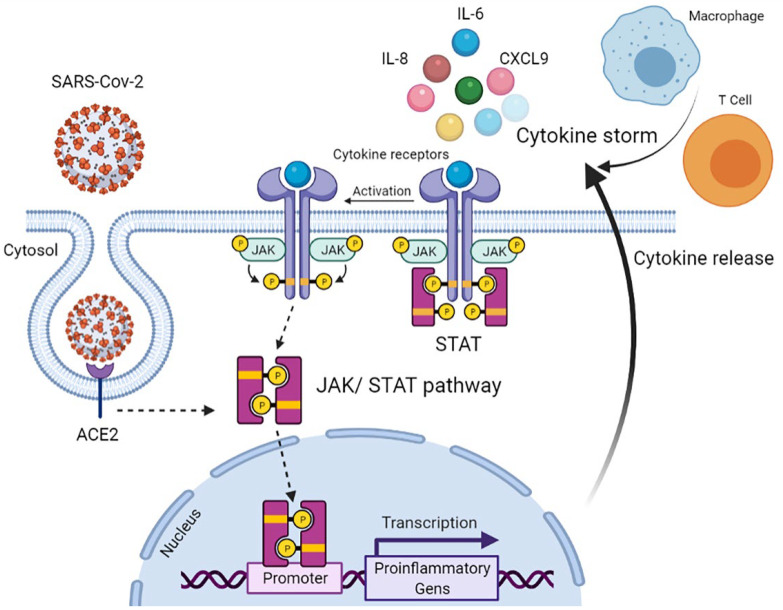
Cytokine release via activation of JAK/STAT signalling pathway following SARS-Cov-2 infection resulting in ARDS related to COVID-19. Abbreviations: ACE2: Angiotensin-converting enzyme 2, CXCL9: Chemokine (C–X–C motif) ligand 9, IL: interleukin, JAK: Janus kinase, and STAT: signal transducer and activator of transcription.

Overall, the Janus kinase (JAK)/signal transducer and the activator of the transcription (STAT) pathway were at the centre of attention for driving hyperinflammation in COVID-19, i.e., the SARS-CoV-2 infection triggers hyperinflammation through the JAK/STAT pathway, resulting in the recruitment of dendritic cells, macrophages, and natural killer (NK) cells, as well as differentiation of B cells and T cells progressing towards cytokine storm [6] (Figure 1).

Unlike the global approach to implementing lockdown to restrain the COVID-19 pandemic, Sweden’s public-health policies were less restrictive.; but when compared to peer countries, Sweden has a higher incidence rate amongst all ages, a death rate in elderly care, and higher all-cause mortality [7]; therefore, we had access to an extensive unique biobank of materials from COVID-19 autopsies in the Pathology Clinic at Karolinska University-Hospital, Stockholm, Sweden. We previously observed that in severe COVID-19 cases, the process of lung consolidation was associated with immature myeloid elements, accumulation of macrophages, and proliferative responses in the stromal and epithelial components [8]. Briefly, we mainly observed an increase in CD163+ myeloid cells in hilar lymph nodes and lung consolidations, as well as a decrease in CD8^+^ T, NK, and B cells in autopsy tissues [8].

Pleural effusion is a pathological term for describing the accumulation of fluid in the pleural space seen during the course of pneumonia [9]. COVID-19 patients with pleural effusion experience longer hospitalization and show multisystem inflammatory syndrome, respiratory failure, ARDS, and higher rates of mortality; therefore, pleural effusion may indicate a poor prognosis for critically ill COVID-19 patients [10] and also as a diagnostic value to differentiate cytokine levels for successful treatment [9].

Following up on our previous report [8], the overall purpose of the current article was to evaluate the inflammatory and organ-damage markers using Olink^®^ panels for pleural effusion samples taken in rapid autopsy from patients who had died of COVID-19.

## 2. Materials and Methods

### 2.1. Study Population

This study comprises 31 individuals, 67% of whom were male. The median age was 65 (ranging from 39 to 94 years old) and the mean body-mass index was 26 ± 3 kg·m^2^. In the COVID-19 cohort (*n* = 29), the cause of death for all patients was determined as COVID-19-associated ARDS (no bacterial superinfection was observed in any cases) and RT-PCR from the nasopharynx or trachea detected the SARS-CoV-2 (details previously published in [8]). The median time between the onset of symptoms and death was 17 days. Non-COVID-19 cohort, as a control (*n* = 2), comprised the pleural effusion of one patient who died of acute myocardial infarction (AMI) and one plasma sample of a healthy alive individual.

### 2.2. Rapid Autopsy and Sample Preparation

Post-mortem pleural effusion fluids were collected during biosafety level 3 autopsies immediately after opening the thoracal cavity. The body was kept in a cold room and a rapid autopsy was performed less than 24 h after death. The fluids were centrifuged at 1800× *g* for 10 min to remove any cellular elements and, then, Triton™ X-100 (Sigma catalogue #9002-93-1) non-ionic, aqueous-solution detergent was added at 0.5% final concentration to inactivate the virus. The effusion fluids were then stored at −20 °C to avoid post-mortem degradation artefacts until further analysis.

### 2.3. Proteomic Analysis: Olink^®^ Proximity Extension Assay (PEA)

The multiplex proximity extension assay is a robust high-throughput protein-marker detection assay showing high accuracy in complex samples, particularly serum and plasma [11]. Olink^®^ PEA technique has been used successfully for COVID-19 analysis repeatedly [12]. The relative abundance of 184 protein biomarkers in pleural effusions was measured using the Olink^®^ Target 96 Inflammation and Olink^®^ Target 96 Organ Damage panels (see Supplementary Table S1), following the manufacturer’s instructions [11]. In brief, each Olink^®^ panel screened 92 proteins using 1 μL of the sample. The Fluidigm BioMark™ HD real-time PCR device was used to quantify the abundance of each protein. Each protein was targeted by a unique pair of probes comprising oligonucleotide-labelled antibodies; hence, anytime two probes were put in proximity, a DNA polymerization resulted in the generation of a particular PCR product that was quantified by real-time PCR. A limit of detection (LOD) was established for each PEA measurement based on negative controls (included in each run) and readings that fell below the LOD were excluded from the analysis. The Olink^®^ PEA data were given as normalized protein expression (NPX) log2 values and were intensity normalized utilizing the plate median, as the normalization factor, for each assay (Intensity Normalization v.2). To eliminate batch effects, samples were distributed randomly throughout the plates. Interplate controls were included on each plate to adjust for plate differences. Biomarkers that did not pass quality control were excluded. For a more detailed description of Olink^®^ PEA technology, assay performance, and validation data please refer to the manufacturer’s protocols at www.olink.com (accessed on the 10 November 2022).

### 2.4. Pearson’s Correlations Coefficient

The samples and the proteins were ordered after hierarchical clustering using the Euclidean distance function. The calculation was conducted using the KNIME 2.9.0 (Konstanz Information Miner, Germany) with a custom-written workflow. More information is available at www.knime.org (accessed on 10 November 2022).

### 2.5. Histology Processing and Immuno-Histochemistry

Samples of lung tissue of all patients were collected in buffer (formaldehyde 4%), subsequently excised in paraffin embedding, and sectioned after fixation. In the accredited routine laboratory of Karolinska Hospital, the blocks were dehydrated and paraffin-embedded using automated tissue processors. Sections (5 μm) were then cut and stained with H&E using automated staining machines. Immunostaining was conducted in Ventana Ultra Benchmark (Ventana Medical Systems, USA), following the manufacturer’s instructions. Immunohistochemistry for PD-L1 and CD8^+^ T-cells was performed using (Roche Cat# 790-4905, RRID: AB_2819099, clone SP263 catalogue # 07494190001, Ventana) and (Santa Cruz Biotechnology Cat# sc-53212, RRID: AB_1120718, clone C8/144B catalogue # M7103, Dako, Agilent), respectively.

### 2.6. Statistical Analysis

The Kolmogorov–Smirnov test was conducted to determine that sampled data from the populations followed a Gaussian (normal) distribution. If the population was following a normal distribution, then a student unpaired (independent), two-tailed t-tPlease add city if availableest was performed to make a comparison between two independent group data (COVID-19 group and non-COVID19 group as control). If the population was not following a normal distribution, then a Wilcoxon signed-rank test was conducted; an α error (*p* < 0.05) level, equal to 95% confidence interval, was used. All statistical tests were performed using GraphPad Prism 8.3.0 (GraphPad Software, San Diego, CA, USA).

## 3. Results

Most of the markers in both Olink^®^ Inflammation and Organ Damage panels were below the detection level; however, the 20 markers showed significant upregulation in deceased COVID-19 patients (Table 1) which can be categorized into two groups based on their function.

**Figure 2:**
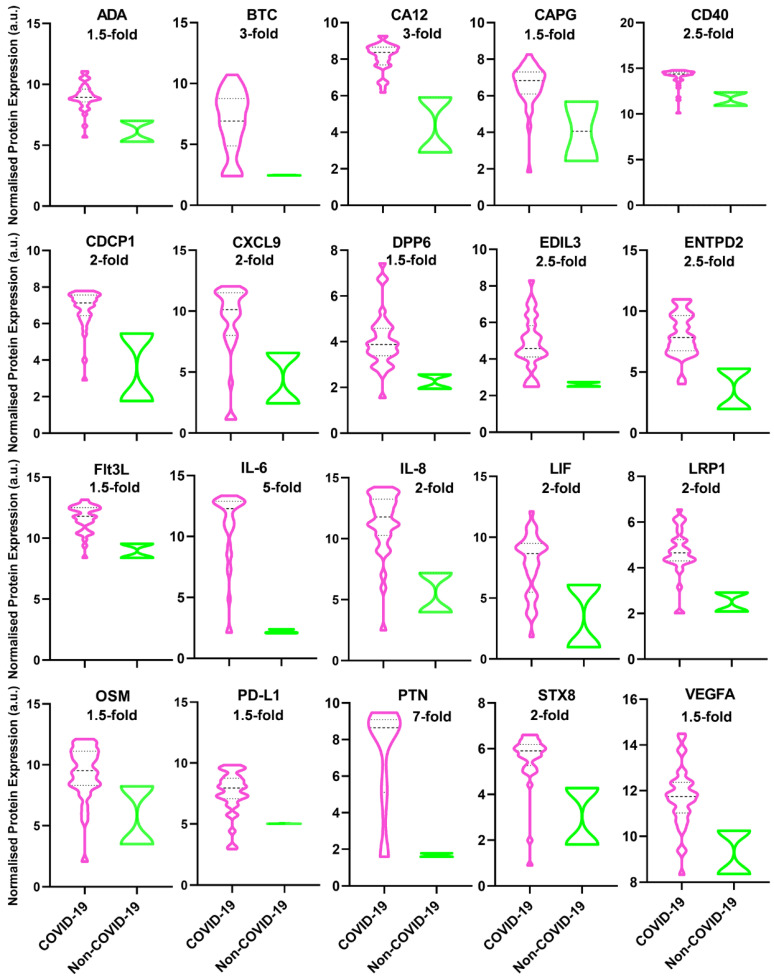
Levels of 20 inflammatory and organ-damage markers were upregulated in deceased COVID-19 patients. All the data presented are statistically significant (*p* < 0.05) compared to the control (non-COVID-19 patients) (COVID-19 cohort = 29, non-COVID-19 cohort = 2, *n* = 2). For the populations that were following normal distribution, a student unpaired t-test was used including BTC, DPP6, EDIL3, ENTPD2, FIt3L, IL-6, OSM, PD-L1, PTN, and VEGFA. For populations that were not following normal distribution, the Wilcoxon signed-rank test was used, including ADA, CA12, CAPG, CD40, CDCP1, CXCL9, IL-8, LIF, LRP1, and STX8. The approximate -fold increase compared to the control has been written for each marker. a.u. arbitrary unit. Note: The description for each marker is summarized in Table 1.

One group of markers show the over-stimulation of the immune system, including ADA, BTC, CA12, CAPG, CD40, CDCP1, CXCL9, ENTPD2, Flt3L, IL-8, LRP1, OSM, PD-L1, PTN, STX8, and VEGFA (Figure 2); particularly, LIF is of interest because indicates negative feedback from the immune system to control cytokine storm.

Another group of markers show arterial and cardiac damage, including DPP6 and EDIL3 (Figure 2).

Conforming with previous reports [3], the level of the following markers, CA12, soluble CD40, IL-6, IL-8, soluble PD-L1, and VEGF, were significantly increased by 1.5-fold, 1.5-fold, 6-fold, 2-fold, 2-fold, and 1.5 fold, respectively, in COVID-19 deceased patients compared to control cohort (*p* ≤ 0.05) (Figure 2).

**Figure 3:**
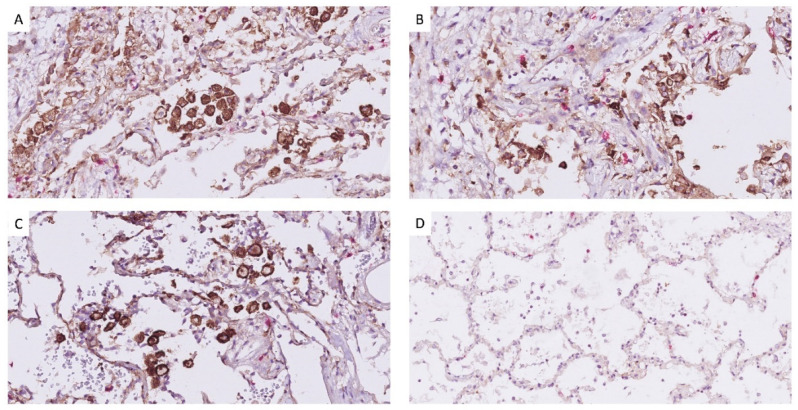
The increased expression of PD-L1 in lung tissue of deceased COVID-19 patients. PD-L1 is expressed on macrophages which are morphologically recognizable (stained in brown) and CD8^+^ T-cells (stained in red). (**A**) patient C10, (**B**) patient C11, (**C**) patient C7, and (**D**) PD-L1 expression is not detected in non-COVID-19 lung control. Note: slide was scanned at 40× resolution using NanoZoomer S360 digital slide-scanner (Hamamatsu, Japan) and visualized by NDP Viewer.

In addition to an increased level of PD-L1 in pleural effusion (Figure 2), the expression of PD-L1 on macrophages also shows enhancement in lung tissues of deceased COVID-19 patients (Figure 3), i.e., the distribution of the PD-L1 staining is similar to CD68 (macrophages), but different from CK18 (epithelial) and CD34 (endothelial), as previously reported by [8].

**Figure 4:**
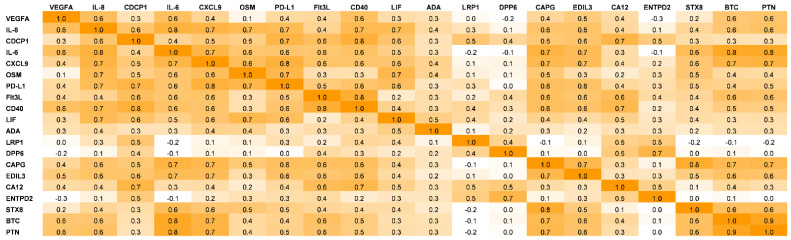
Correlation matrix of raised markers in deceased COVID-19 patients. Pearson’s correlation coefficient is a statistical measure that expresses the strength of a linear relationship between two paired sets of data. The background colours mark the value of the coefficient (orange is significant but white is non-significant); the positive correlation between paired data is “0.8 ≤ *r* ≤ 1.0 = very strong”, “0.6 ≤ *r* ≤ 0.79 = strong”, “0.4 ≤ *r* ≤ 0.59 = moderate”,“ 0.20 ≤ *r* ≤ 0.39 = weak”, and “0.0 ≤ *r* ≤ 0.19 = very weak”.

The use of Pearson’s correlations coefficient shows a very strong correlation between sets of proteins, including (Flt3L/sCD40), (BTC/PTN), (IL-6/IL-8/VEGFA), (IL-6/BTC/PTN), (CAPG/EDIL3), and (CXCL9/OSM/PD-L1) (Figure 4).

## 4. Discussion

To the best of our knowledge, this is the first time that markers ADA, BTC, DPP6, EDIL3, ENTPD2, Flt3L, LIF, and LRP1 are reported to be upregulated in severe COVID-19 patients; hence, they can be proposed as potential biomarkers for severe cases of COVID-19; for example, LIF is not normally produced in the lungs, but when alveolar macrophages, while patrolling the blood–air barrier, encounter a virus, they release inflammatory cytokines as an alarm, which triggers LIF production. LIF protects alveolar type I and II cells, preventing scarring, fibrosis, and the collapse of the air niche [33]. LIF has been identified as a lung-protecting agent in animal models of pneumonia because it prevents severe disease development. LIF levels are increased in COVID-19, but no data have been reported yet [34]. Because LIF is a lung-protective agent, it has been proposed that recombinant LIF be administered to protect the lung during COVID-19. Given its safety in phase I and II clinical trials, the rationale is to prevent severe forms and long-term disease [35]; hence, our result showing a two-fold increase in LIF highlights the rationale of using recombinant LIF in COVID-19 patients.

Conforming with previous reports which were mostly conducted using the peripheral blood of COVID-19 patients [3], our study shows a significant increase in the following markers, CA12, CAPG, CDPC1, CD40, CXCL9, IL-6, IL-8, OSM, PD-L1, PTN, STX8, and VEGF, thus, pinpointing their relevance to be applied for the treatment of critically ill COVID-19 patients (Figure 2). The following markers and treatments can be recommended according to our results.

COVID-19 patients demonstrate a dysregulated acid-base status affected by increased CA activity. It has been demonstrated that dexamethasone decreases CA9 expression via the HIF-1α-dependent mechanism; thus, classical CA inhibitors (e.g., acetazolamide and methazolamide) could be used as an adjunctive treatment for COVID-19 patients; for example, acetazolamide treatment improved respiratory conditions in COVID-19 patients in a clinical study [15].

Furthermore, platelets are hyperresponsive, more adhesive, and stimulated to release inflammatory cytokines leading to thrombo-inflammation in COVID-19 [36]; as a consequence, soluble CD40 Ligand, as a plasmatic marker for platelet aggregation, was reported to be enhanced in deceased patients compared to recovered COVID-19 patients [36], suggesting CD40 as a prognostic marker for thrombo-inflammation in severe COVID-19 cases.

Despite encouraging recoveries with anti-IL-6 drugs (tocilizumab and sarilumab), tocilizumab, particularly, showed mixed results in terms of clinical benefit in single-arm studies and early randomized trials; however, clinical trials (e.g., RECOVERY and REMAP-CAP) are ongoing and applying a combination of anti-IL-6 with steroids. It is proposed that the best benefit of anti-IL-6 therapies may necessitate early intervention at the onset of hyperinflammation but before the virus causes irreversible damage to tissues [24]. In general, anti-IL6 agents appear to be effective against medium to severe cases of COVID-19, increasing CD8^+^ T-cell frequency and functionalities, preventing multi-organ failure, reducing inflammation levels, and, thus, reducing the risk of mortality [24].

Levels of IL-8 more reliably predict the overall clinical COVID-19 disease scores at different stages than IL-6 levels; therefore, IL-8 has been suggested as a biomarker for the severity and prognosis of COVID-19 cases [25]. In addition, a randomized Phase II clinical trial is ongoing, using anti-IL-8 therapy compared to the standard of care in severe COVID-19 patients (ClinicalTrials.gov Identifier: NCT04347226 accessed on 30 November 2021).

It has been shown that in severe COVID-19 patients, T lymphocytes are switching from hyperactivated status to exhaustion, expressing higher levels of PD-L1; however, the data on the importance of PD-L1 dysregulation during SARS-CoV-2 infection are yet inconclusive and our knowledge is currently limited on the role of soluble PD-L1 in COVID-19 based on the different grades of severity and prognosis [37]. In addition, COVID-19 patients who were treated with anti-PD-L1 and anti-PD-1 antibodies restored their T-lymphocytes’ competence and efficaciously fought off the infection. To this date, four clinical trials are ongoing to investigate the efficacy of anti-PD-1 antibodies in both groups of cancer and non-cancer patients infected with SARS-Cov-2. The results may suggest that restoring exhausted T lymphocytes is a potential treatment [38].

Hypoxia induces VEGF expression by activating the hypoxia-inducible factor-1 pathway which participates in vascular dysfunction and inflammation of the lungs [32]. A single-arm trial showed that blocking VEGF and the VEGF receptor-mediated signalling using bevacizumab, a humanized anti-VEGF monoclonal antibody, improves anti-inflammatory response and oxygen perfusion, alleviating clinical symptoms in critically ill COVID-19 patients [32].

The following interpretation can be given based on Pearson’s correlation coefficient analysis.

BTC/PTN: These two proteins engage in the induction of the inflammatory microenvironment (Table 1).

CXCL9/OSM/PD-L1: According to bioinformatics database (Cancer Genome Atlas and Gene Expression Omnibus) analysis which was then confirmed by in vitro and in vivo experiments, PD-L1 expression is upregulated by CXCL9-11 through activation of the STAT/PI3K-Akt pathways [39]. Interestingly, OSM also binds to the OSM receptor, leading to the recruitment of JAKs and subsequent signal transduction and activation of STAT3 [40]; thus, we can hypothesize that both CXCL9 and OSM participate in the upregulation of PD-L1 through the STAT activation pathway in COVID-19 patients (Figure 1).

IL-6/IL-8/VEGFA: The production of the triad (IL-6, IL-8, and VEGF) is reportedly induced through STAT1 signalling in lung cancer [41]; hence, the simultaneous increase in IL-6/IL-8/VEGFA and CXCL9/OSM/PD-L1 in our study could propose the possible involvement of the JAK/STAT signalling pathway as a core regulator of hyperinflammation in COVID-19 patients. Currently, there are tens of clinical trials for applications of JAK inhibitors (e.g., Baricitinib Fedratinib, Oclacitinib, Ruxolitinib, Tofacitinib, and Upadacitinib) in COVID-19 patients either as single or combinatorial therapies. More information has been provided in the following review articles [6,42].

CAPG/EDIL3: CAPG is a biomarker for macrophage activation (Table 1). EDIL3 plays a key role, particularly in leukocyte recruitment and adhesion to the endothelium (Table 1), i.e., EDIL3 acts as an anti-adhesive factor, consequently, preventing leukocyte adhesion to the endothelium [43]; thus, we can hypothesize that the associated upregulation of CAPG/EDIL3 leads to activation and non-adhesivity of macrophages in lung tissues.

CD40/Flt3L: It has already been shown that CD40 ligand, through upregulation of Flt3L and thrombopoietin, stimulates myelopoiesis and, subsequently, triggers myeloid cells (e.g., dendritic cells, macrophages, and monocytes) to release cytokines, such as IL-6, IL-8, or LIF [44].

Therefore, our result, conforming with previous reports, indicates that simultaneous upregulation of CD40, Flt3L, IL-6, IL-8, and LIF (Figure 3), in association with CAPG and EDIL3 (Figure 3), plays a pivotal role in inflammation and recruitment of immune cells in lung tissues of COVID-19 patients.

### Limitations of the Study

To the best of our knowledge, this is the first study using autopsy specimens of COVID-19 patients for assessing a wide array of inflammatory and organ-damage markers; however, most of the markers were either below the detection level or were not significantly different compared to the control but our results confirm the previous reports about hyperinflammation in COVID-19. Furthermore, this is a single-centre study using patients deceased of COVID-19 complications; yet, our results can be verified in a larger multi-centre scenario. Likewise, we included severe cases of COVID-19 patients who died of ARDS-associated symptoms; thus, these data do not apply to asymptomatic or mild cases of COVID-19.

Generally, proteins are known to degrade post-mortem; to mitigate this risk, a strategy for rapid autopsy was undertaken. Another limitation of our study is that we had access to only a small amount of control due to the limitations caused by autopsy materials; thus, only the values of markers that were significantly higher than the control were included in this analysis but the values which were lower compared to control were excluded.

## 5. Conclusions

In summary, the samples of 31 individuals (29 pleural effusions of deceased COVID-19 patients, and 2 non-COVID-19 controls) were analyzed using Olink^®^ Inflammation and Organ Damage panels. Out of 184 protein markers, 20 markers were raised significantly in COVID-19 deceased patients. A group of markers showed over-stimulation of the immune system, including ADA, BTC, CA12, CAPG, CD40, CDCP1, CXCL9, ENTPD2, Flt3L, IL-6, IL-8, LRP1, OSM, PD-L1, PTN, STX8, and VEGFA; furthermore, DPP6 and EDIL3 indicated damage to arterial and cardiovascular organs. Conforming with previous studies which mostly used peripheral blood samples, our pleural effusions study also confirmed the elevated levels of CA12, CD40, IL-6, IL-8, PD-L1, and VEGFA, proposing their potential either as biomarkers for severity and prognosis of the disease or targets for therapy. Furthermore, this study reports markers including ADA, BTC, DPP6, EDIL3, LIF, ENTPD2, Flt3L, and LRP1 to be upregulated in severe COVID-19 patients; hence, they are proposed as novel biomarkers for severe cases of COVID-19. In addition, Pearson’s correlation coefficient analysis suggested the involvement of JAK/STAT pathways in cytokine storm and hyperinflammation in deceased COVID-19 patients; thus, the pursuit of studies about the possible application of JAK inhibitors against COVID-19 is recommended in future studies. The analysis of identified markers using in silico models also is recommended in future to predict a more accurate image of how the signalling pathway might help combat COVID-19 severe cases.

## Figures and Tables

**Table 1:** A list of the markers showed significant upregulation in COVID-19 deceased patients.

Abbreviation	Protein Name	Function
ADA	Adenosine deaminase	An enzyme converts adenosine to inosine, vital for the differentiation of lymphoid cells while showing enhanced activity in diseases wherein immunity is stimulated [13].
BTC	Betacellulin	A member of the EGF family binds to ErbB1 and ErbB4 homodimers, activating the EGFR–PI3K–Akt–Erk pathway to form an inflammatory microenvironment and produce IL-8 [14].
CA12	Carbonic anhydrase 12	An enzyme catalyzes the hydration of carbon dioxide to bicarbonate and H^+^ ions, involved in many physiological processes. The similarity between high-altitude pulmonary oedema and COVID-19 suggests the possible role of CA in COVID-19, thus proposing the use of CA inhibitors for COVID-19 treatment [15].
CAPG	Macrophage-capping protein	A pro-inflammatory mediator released by macrophage activation subsequently triggers pro-inflammatory cytokine release. The overexpression of CAPG also reveals the ongoing state of inflammatory diseases [16].
CD40	CD40	CD40 and CD40L are surface receptors, members of the TNF and TNF receptor superfamilies, respectively. Inducing inflammatory and pro-thrombotic responses. CD40 is expressed on monocytes, macrophages, B, T, NK, and dendritic cells. In addition to platelet activation, CD40/CD40L interaction regulates a variety of cellular and molecular processes involved in innate and adaptive immune responses [17].
CDCP1	CUB domain-containing protein 1	Typically, a key regulator of tumour cell survival and metastasis, affecting immune-mediated diseases; however, CDCP1 is one of the most upregulated genes in SARS-CoV-2-infected children with Kawasaki disease [18].
CXCL9	C-X-C motif chemokine ligand 9	One of the major chemokines reported being elevated in SARS-CoV infection [19].
DPP6	Dipeptidyl aminopeptidase-like protein 6	DPP6 overexpression in Purkinje fibres elicits short-coupled extrasystoles triggering idiopathic ventricular fibrillation [20].
EDIL3	EGF-like repeat and discoidin I-like domain-containing protein 3	A glycoprotein in arterial vessel walls which is overexpressed in Kawasaki disease or after vascular injury; also, it inhibits the recruitment and extravasation of inflammatory cells across the endothelium [21].
ENTPD2	Ectonucleoside triphosphate diphosphohydrolase 2	Expression of ENTPD2 in the enteric nervous system exacerbates inflammation in inflammatory bowel disease [22].
Flt3L	Fms-related tyrosine kinase 3 ligand	Flt3L functions as both a cytokine and a growth factor, increasing the amount of immune cells, especially dendritic cells and lymphocytes [23].
IL-6	Interleukin 6	High IL-6 has been linked consistently to severe COVID-19 cases [24].
IL-8	Interleukin 8	A strong biomarker for the severity of COVID-19 and disease prognosis [25].
LIF	Leukaemia inhibitory factor	In viral pneumonia, LIF opposes the cytokine storm in the lungs [26].
LRP1	Prolow-density lipoprotein receptor-related protein 1	A modulator of tissue inflammation and organ repair, e.g., the brain, kidney, lung, vasculature, and AMI [27].
OSM	Oncostatin-M	A key regulator of IL-6 and a biomarker for the clinical severity of COVID-19, suggesting the role of bacterial product infection [28].
PD-L1	Programmed cell death 1 ligand 1	PD-L1 is overexpressed in monocytes, NK cells, and, particularly, basophils and eosinophils in severe COVID-19 patients. The cytokine storm may be led to increased PD-L1 expression, leading to CD8^+^ T-cell exhaustion. Blockading PD-L1/PD-1 could recover CD8^+^ T-cell numbers and functionalities [29].
PTN	Pleiotrophin	A small cationic protein which is associated with bone development, cancer metastasis, inflammation, neural regeneration, and tissue repair [30].
STX8	Syntaxin-8	Vesicle trafficking protein is necessary for lytic granule trafficking in cytotoxic T lymphocytes [31].
VEGFA	Vascular Endothelial Growth Factor A	Hypoxia upregulates VEGF expression inducing vascular leakiness in SARS-Cov-2-infected lung tissues, resulting in plasma extravasation and pulmonary oedema, later increasing tissue hypoxia in a vicious cycle [32].

## Supplementary Materials

The following supporting information can be downloaded at: www.mdpi.com/article/10.3390/diagnostics12112789/s1, Table S1: List of 184 protein biomarkers in the Olink® Target 96 Inflammation and Olink® Target 96 Organ Damage panels.
