# Supplementary material for: Proteomic Analysis of Pleural Effusions from COVID-19 Deceased Patients: Enhanced Inflammatory Markers
Source: Diagnostics (Basel). 2022 Nov 14;12(11):2789. doi: 10.3390/diagnostics12112789 (PMC9689825; doi:10.3390/diagnostics12112789)
Supplement: Supplementary file 1 [file diagnostics-12-02789-s001.zip › diagnostics-1968835-supplementary.pdf]

Supplementary Table S1. List of 184 protein biomarkers in the Olink® Target 96 Inflammation and Olink® Target 96 Organ Damage panels

Protein Name	Gene Name	Uniprot ID
Adhesion G-protein coupled receptor G1	ADGRG1	Q9Y653
Anterior gradient protein 2 homolog (AG-2)	AGR2	O95994
Apoptosis-inducing factor 1, mitochondrial	AIFM1	O95831
Aldehyde dehydrogenase, dimeric NADP-preferring	ALDH3A1	P30838
Protein amnionless	AMN	Q9BXJ7
Renin receptor	ATP6AP2	O75787
BMP and activin membrane-bound inhibitor homolog	BAMBI	Q13145
B-cell scaffold protein with ankyrin repeats	BANK1	Q8NDB2
BH3-interacting domain death agonist	BID	P55957
Probetacellulin	BTC	P35070
Carbonic anhydrase 12	CA12	O43570
Carbonic anhydrase 14	CA14	Q9ULX7
Calcitonin	CALCA	P01258
Calreticulin	CALR	P27797
Macrophage-capping protein	CAPG	P40121
Cocaine esterase	CES2	O00748
C-type lectin domain family 1 member A	CLEC1A	Q8NC01
Claspin (hClaspin)	CLSPN	Q9HAW4
Contactin-2	CNTN2	Q02246
Corticoliberin	CRH	P06850
Casein kinase I isoform delta (CKI-delta)	CSNK1D	P48730
Dipeptidyl aminopeptidase-like protein 6	DPP6	P42658
Desmoglein-4	DSG4	Q86SJ6
EGF-like repeat and discoidin I-like domain-containing protein 3	EDIL3	O43854
Epidermal growth factor-like protein 7 (EGF-like protein 7)	EGFL7	Q9UHF1
Protein enabled homolog	ENAH	Q8N8S7
Ectonucleoside triphosphate diphosphohydrolase 2 (NTPDase 2)	ENTPD2	Q9Y5L3
Ectonucleoside triphosphate diphosphohydrolase 6 (NTPDase 6)	ENTPD6	O75354
Erythropoietin	EPO	P01588
Protein LAP2	ERBB2IP	Q96RT1
Fatty acid-binding protein 9	FABP9	Q0Z7S8
Tyrosine-protein kinase Fes/Fps	FES	P07332
Tyrosine-protein kinase Fgr	FGR	P09769
Peptidyl-prolyl cis-trans isomerase FKBP1B (PPIase FKBP1B)	FKBP1B	P68106
Protein fosB	FOSB	P53539
Forkhead box protein O1	FOXO1	Q12778
Polypeptide N-acetylgalactosaminyltransferase 10	GALNT10	Q86SR1
Hematopoietic prostaglandin D synthase (H-PGDS)	HPGDS	O60760
Phosphatidylinositol 3,4,5-trisphosphate 5-phosphatase 2	INPPL1	O15357
Integrin beta-1-binding protein 1	ITGB1BP1	O14713
Kidney Injury Molecule	KIM1	Q96D42
Killer cell immunoglobulin-like receptor 3DL1	KIR3DL1	P43629
Linker for activation of T-cells family member 2	LAT2	Q9GZY6
Lutropin subunit beta (Lutropin beta chain)	LHB	P01229
Prolow-density lipoprotein receptor-related protein 1 (LRP-1)	LRP1	Q07954

Leukotriene A-4 hydrolase (LTA-4 hydrolase)	LTA4H	P09960
Macrophage erythroblast attacher	MAEA	Q7L5Y9
Melanoma-associated antigen D1	MAGED1	Q9Y5V3
Mitogen-activated protein kinase kinase kinase kinase 5	MAP4K5	Q9Y4K4
Protein max	MAX	P61244
Methionine aminopeptidase 1 (MAP 1)	METAP1	P53582
Mevalonate kinase (MK)	MVK	Q03426
Nibrin	NBN	O60934
Neutrophil cytosol factor 2 (NCF-2)	NCF2	P19878
Nitric oxide synthase, endothelial	NOS3	P29474
C-type natriuretic peptide	NPPC	P23582
NEDD8 ultimate buster 1	NUB1	Q9Y5A7
Nucleobindin-2	NUCB2	P80303
Programmed cell death protein 1 (Protein PD-1)	PDCD1	Q15116
Platelet-derived growth factor C (PDGF-C)	PDGFC	Q9NRA1
[Pyruvate dehydrogenase [acetyl-transferring]]-phosphatase 1, mitochondrial (PDP 1)	PDP1	Q9P0J1
Placenta growth factor (PlGF)	PGF	P49763
Perilipin-1	PLIN1	O60240
Plexin domain-containing protein 1	PLXDC1	Q8IUK5
Serum paraoxonase/arylesterase 2 (PON 2)	PON2	Q15165
Protein phosphatase 1B	PPM1B	O75688
5'-AMP-activated protein kinase subunit beta-1 (AMPK subunit beta-1)	PRKAB1	Q9Y478
Interferon-inducible double-stranded RNA-dependent protein kinase activator A	PRKRA	O75569
Proteasome subunit alpha type-1	PSMA1	P25786
Inactive tyrosine-protein kinase 7	PTK7	Q13308
Pleiotrophin (PTN)	PTN	P21246
Receptor-type tyrosine-protein phosphatase eta (Protein-tyrosine phosphatase eta)	PTPRJ	Q12913
Parvalbumin alpha	PVALB	P20472
Paxillin	PXN	P49023
Retinoic acid receptor responder protein 1	RARRES1	P49788
Ras GTPase-activating protein 1 (GAP)	RASA1	P20936
Ras association domain-containing protein 2	RASSF2	P50749
REST corepressor 1	RCOR1	Q9UKL0
Ribonucleoside-diphosphate reductase subunit M2 B	RRM2B	Q7LG56
Serpin A9	SERPINA9	Q86WD7
NAD-dependent protein deacylase sirtuin-5, mitochondrial	SIRT5	Q9NXA8
Mothers against decapentaplegic homolog 1 (MAD homolog 1)	SMAD1	Q15797
CMP-N-acetylneuraminate-beta-galactosamide-alpha-2,3-sialyltransferase 1 (Alpha 2,3-ST 1)	ST3GAL1	Q11201
Syntaxin-8	STX8	Q9UNK0
Syntaxin-binding protein 3	STXBP3	O00186
Fructose-2,6-bisphosphatase TIGAR	TIGAR	Q9NQ88
Enteropeptidase	TMPRSS15	P98073
Troponin I, cardiac muscle	TNNI3	P19429
DNA topoisomerase 2-beta	TOP2B	Q02880
Vasohibin-1	VASH1	Q7L8A9

Wiskott-Aldrich syndrome protein (WASp)	WAS	P42768
Tyrosine-protein kinase Yes	YES1	P07947
Eukaryotic translation initiation factor 4E-binding protein 1	4E-BP1	Q13541
Adenosine Deaminase	ADA	P00813
Artemin	ARTN	Q5T4W7
Axin-1	AXIN1	O15169
Beta-nerve growth factor	Beta-NGF	P01138
Caspase 8	CASP-8	Q14790
Eotaxin-1	CCL11	P51671
C-C motif chemokine 19	CCL19	Q99731
C-C motif chemokine 20	CCL20	P78556
C-C motif chemokine 23	CCL23	P55773
C-C motif chemokine 25	CCL25	O15444
C-C motif chemokine 28	CCL28	Q9NRJ3
Macrophage inflammatory protein 1-alpha	CCL3	P10147
C-C motif chemokine 4	CCL4	P13236
Natural killer cell receptor 2B4	CD244	Q9BZW8
CD40L receptor	CD40	P25942
T-cell surface glycoprotein CD5	CD5	P06127
T cell surface glycoprotein CD6 isoform	CD6	P30203
T-cell surface glycoprotein CD8 alpha chain	CD8A	P01732
CUB domain-containing protein 1	CDCP1	Q9H5V8
Macrophage colony-stimulating factor 1	CSF-1	P09603
Cystatin D	CST5	P28325
Fractalkine	CX3CL1	P78423
C-X-C motif chemokine 1	CXCL1	P09341
C-X-C motif chemokine 10	CXCL10	P02778
C-X-C motif chemokine 11	CXCL11	O14625
C-X-C motif chemokine 5	CXCL5	P42830
C-X-C motif chemokine 6	CXCL6	P80162
C-X-C motif chemokine 9	CXCL9	Q07325
Delta and Notch-like epidermal growth factor-related receptor	DNER	Q8NFT8
Protein S100-A12	EN-RAGE	P80511
Fibroblast growth factor 19	FGF-19	O95750
Fibroblast growth factor 21	FGF-21	Q9NSA1
Fibroblast growth factor 23	FGF-23	Q9GZV9
Fibroblast growth factor 5	FGF-5	P12034
Fms-related tyrosine kinase 3 ligand	Flt3L	P49771
Glial cell line-derived neurotrophic factor	GDNF	P39905
Hepatocyte growth factor	HGF	P14210
Interferon gamma	IFN-gamma	P01579
Interleukin-1 alpha	IL-1 alpha	P01583
Interleukin-10	IL10	P22301
Interleukin-10 receptor subunit alpha	IL-10RA	Q13651
Interleukin-10 receptor subunit beta	IL-10RB	Q08334
Interleukin-12 subunit beta	IL-12B	P29460
Interleukin-13	IL13	P35225
Interleukin-15 receptor subunit alpha	IL-15RA	Q13261

Interleukin-17A	IL-17A	Q16552
Interleukin-17C	IL-17C	Q9P0M4
Interleukin-18	IL18	Q14116
Interleukin-18 receptor 1	IL-18R1	Q13478
Interleukin-2	IL2	P60568
Interleukin-20	IL-20	Q9NYY1
Interleukin-20 receptor subunit alpha	IL-20RA	Q9UHF4
Interleukin-22 receptor subunit alpha-1	IL-22 RA1	Q8N6P7
Interleukin-24	IL-24	Q13007
Interleukin-2 receptor subunit beta	IL-2RB	P14784
Interleukin-33	IL33	O95760
Interleukin-4	IL4	P05112
Interleukin-5	IL5	P05113
Interleukin-6	IL6	P05231
Interleukin-7	IL7	P13232
Interleukin-8	IL8	P10145
Latency-associated peptide transforming growth factor beta 1	LAP TGF-beta-1	P01137
Leukemia inhibitory factor	LIF	P15018
Leukemia inhibitory factor receptor	LIF-R	P42702
Monocyte chemotactic protein 1	MCP-1	P13500
Monocyte chemotactic protein 2	MCP-2	P80075
Monocyte chemotactic protein 3	MCP-3	P80098
Monocyte chemotactic protein 4	MCP-4	Q99616
Matrix metalloproteinase-1	MMP-1	P03956
Matrix metalloproteinase-10	MMP-10	P09238
Neurturin	NRTN	Q99748
Neurotrophin-3	NT-3	P20783
Osteoprotegerin	OPG	O00300
Oncostatin-M	OSM	P13725
Programmed cell death 1 ligand 1	PD-L1	Q9NZQ7
Stem cell factor	SCF	P21583
SIR2-like protein 2	SIRT2	Q8IXJ6
Signaling lymphocytic activation molecule	SLAMF1	Q13291
Sulfotransferase 1A1	ST1A1	P50225
STAM-binding protein	STAMBP	O95630
Transforming growth factor alpha	TGF-alpha	P01135
Tumor necrosis factor	TNF	P01375
TNF-beta	TNFB	P01374
Tumor necrosis factor receptor superfamily member 9	TNFRSF9	Q07011
Tumor necrosis factor ligand superfamily member 14	TNFSF14	O43557
TNF-related apoptosis-inducing ligand	TRAIL	P50591
TNF-related activation-induced cytokine	TRANCE	O14788
Thymic stromal lymphopoietin	TSLP	Q969D9
Tumor necrosis factor (Ligand) superfamily, member 12	TWEAK	O43508
Urokinase-type plasminogen activator	uPA	P00749
Vascular endothelial growth factor A	VEGFA	P15692
